# The Promise of the Zebrafish Model for Parkinson’s Disease: Today’s Science and Tomorrow’s Treatment

**DOI:** 10.3389/fgene.2021.655550

**Published:** 2021-04-15

**Authors:** Khairiah Razali, Noratikah Othman, Mohd Hamzah Mohd Nasir, Abd Almonem Doolaanea, Jaya Kumar, Wisam Nabeel Ibrahim, Norlinah Mohamed Ibrahim, Wael M. Y. Mohamed

**Affiliations:** ^1^Department of Basic Medical Sciences, Kulliyyah of Medicine, International Islamic University Malaysia (IIUM), Kuantan, Malaysia; ^2^Department of Basic Medical Sciences, Kulliyyah of Nursing, International Islamic University Malaysia (IIUM), Kuantan, Malaysia; ^3^Central Research and Animal Facility (CREAM), Kulliyyah of Science, International Islamic University Malaysia (IIUM), Kuantan, Malaysia; ^4^Department of Pharmaceutical Technology, Kulliyyah of Pharmacy, International Islamic University Malaysia (IIUM), Kuantan, Malaysia; ^5^Department of Physiology, Faculty of Medicine, UKM Medical Centre (UKMMC), Kuala Lumpur, Malaysia; ^6^Department of Biomedical Sciences, College of Health Sciences, QU Health, Qatar University, Doha, Qatar; ^7^Biomedical and Pharmaceutical Research Unit, QU Health, Qatar University, Doha, Qatar; ^8^Neurology Unit, Department of Medicine, UKM Medical Centre (UKMMC), Kuala Lumpur, Malaysia; ^9^Clinical Pharmacology Department, Menoufia Medical School, Menoufia University, Menoufia, Egypt

**Keywords:** zebrafish, neurodegenerative diseases, Parkinson’s disease, dopaminergic neuron, MPTP, animal model

## Abstract

The second most prevalent neurodegenerative disorder in the elderly is Parkinson’s disease (PD). Its etiology is unclear and there are no available disease-modifying medicines. Therefore, more evidence is required concerning its pathogenesis. The use of the neurotoxin 1-methyl-4-phenyl-1,2,3,6-tetrahydropyridine (MPTP) is the basis of most animal models of PD. MPTP is metabolized by monoamine oxidase B (MAO B) to MPP + and induces the loss of dopaminergic neurons in the substantia nigra in mammals. Zebrafish have been commonly used in developmental biology as a model organism, but owing to its perfect mix of properties, it is now emerging as a model for human diseases. Zebrafish (*Danio rerio*) are cheap and easy to sustain, evolve rapidly, breed transparent embryos in large amounts, and are readily manipulated by different methods, particularly genetic ones. Furthermore, zebrafish are vertebrate species and mammalian findings obtained from zebrafish may be more applicable than those derived from genetic models of invertebrates such as *Drosophila melanogaster* and *Caenorhabditis elegans*. The resemblance cannot be taken for granted, however. The goal of the present review article is to highlight the promise of zebrafish as a PD animal model. As its aminergic structures, MPTP mode of action, and PINK1 roles mimic those of mammalians, zebrafish seems to be a viable model for studying PD. The roles of zebrafish MAO, however, vary from those of the two types of MAO present in mammals. The benefits unique to zebrafish, such as the ability to perform large-scale genetic or drug screens, should be exploited in future experiments utilizing zebrafish PD models.

## Introduction

Preclinical research is the gateway to the more complicated and ever-challenging clinical or human research (Huang et al., 2020). Concerning this, translational medicine is a discipline that provides the interconnectivity between preclinical and clinical research, the interpretation of preclinical outcomes, and its application into clinical studies (Cohrs et al., 2015; Jacques and Suuronen, 2020). Preclinical research is important especially for drug discoveries and treatment efficiencies to ensure their safety and efficacy before being applied to humans (Regenberg et al., 2009; Varga et al., 2010; Park and Schaer, 2019). In choosing an animal model for preclinical research, the animal species must have adequate information accessibility, be easily managed (tractable), and has comparative and translational potentials (Dietrich et al., 2020). Generally, an animal species is defined as a model organism based on its comparable physiology and anatomy, genetic homogeneity, and response similarly to treatments as humans (Barré-Sinoussi and Montagutelli, 2015; Swearengen, 2018). Over the centuries, researchers have introduced various animal species suitable to be used as study models in scientific research. Examples of the animals are roundworms (*Caenorhabditis elegans*), fruit flies (*Drosophila melanogaster*), zebrafish (*Danio rerio*), rodents (*Mus musculus* and *Rattus norvegicus*), and non-human primates (Chia et al., 2020).

In neuroscience research, human-based studies are challenging and limited, mainly because most experiments need to be done on the brain *in vitro* (which is only applicable through post-mortem) (Shamoo, 2010; Gomez-Nicola and Boche, 2015; Budday et al., 2019). Hence, animals that have significant functional similarities with humans such as fruit flies, rodents, and zebrafish provide excellent alternatives to conduct comprehensive studies on the nervous system. Danio rerio, or more commonly known as zebrafish, has been a model for various neuroscience-related studies that were established 3–4 decades ago (Rahman Khan and Sulaiman Alhewairini, 2019). This particular species is a freshwater teleost that falls under the Cyprinidae family. Over the years, researchers have performed rapid development of methodologies and techniques that allow optimal application of zebrafish in neuroscience research (Saleem and Kannan, 2018). With its close neurofunctional and behavioral similarities to humans, zebrafish have become an excellent model of neurodegenerative, neurodevelopmental, and neuropharmacological studies (Best and Alderton, 2008; Vaz et al., 2018). Moreover, its well-characterized nervous system has led zebrafish to become a suitable model, possibly replacing rodent models, for studying PD, the second most common neurodegenerative disease after Alzheimer’s disease.

Even though extensive information is available regarding the epidemiology and possible managements of PD, researchers are still overwhelmed by its complexity and the challenges faced to cure this disease (DeMaagd and Philip, 2015; Pirtošek et al., 2020). Hence, this review focuses on the use of zebrafish in neuroscience research, particularly those relating to PD studies. Since the past decades up until now, zebrafish have gracefully carved their way in becoming an animal model of PD and provides a promising future in combating this chronic and progressive neurodegenerative disease.

## Parkinson’s Disease

PD is a type of neurological disease typically associated with motor symptoms that results from progressive degeneration of the dopaminergic (DA) neurons in the substantia nigra pars compacta (SNpc) (DeMaagd and Philip, 2015). The 2016 Global Burden of Disease Study showed that the prevalence and death rates of PD had increased significantly over the past 25 years and are projected to rise further (Feigin et al., 2017; Ray Dorsey et al., 2018). Compared to 6.1 million reported PD patients in 2016, currently, there are more than 10 million PD patients worldwide (Ray Dorsey et al., 2018) and it is estimated that the case will reach 12 million by 2050 (Rocca, 2018). The mortality of PD is believed to be caused mainly by aspiration pneumonia, a lung infection that is possibly due to the aspiration of substances from the stomach into the lungs (Suttrup and Warnecke, 2016; Hobson and Meara, 2018). This is supported by the fact that as PD progresses to the later stage, it severely affects the ability to coordinate between swallowing and breathing processes, a condition known as oropharyngeal dysphagia (Suttrup and Warnecke, 2016). Other reported complications of PD that lead to death include cerebrovascular and cardiovascular diseases (Pinter et al., 2015).

### Etiology and Pathogenesis of PD

Decades of clinical and non-clinical PD studies have unveiled several environmental and genetic factors that are contributory to the rising of PD, but increased longevity is perhaps the most obvious contributing factor (Reeve et al., 2014; Ball et al., 2019). It is estimated that 96% of individuals diagnosed with PD are above the age of 60 years (Lee et al., 2019). Several toxic environmental agents increase the risk of developing PD symptoms, such as 1-methyl-4-phenyl-1,2,3,6-tetrahydropyridine (MPTP), 6-hydroxydopamine (6-OHDA), rotenone, and paraquat (Nonnekes et al., 2018; Bastías-Candia et al., 2019). Apart from the environmental factors, mutation to certain genes also increases the susceptibility to PD. For instance, mutations in the α-synuclein (*SNCA*) and *LRRK2* genes cause autosomal dominant PD while *PARK2, PINK1*, and *PARK7* gene mutations cause autosomal recessive PD (Billingsley et al., 2018; Pang et al., 2019). The complex interplay between genetic and environmental factors challenges the course of finding the cure to this disease.

Parkinson’s disease affects the population of DA neurons in SNpc and since this particular brain region is the origin of the nigrostriatal dopaminergic system, gradual loss of DA neurons in SNpc results in subsequent loss of dopamine input into the striatum (Sarath Babu et al., 2016; Barnhill et al., 2020), causing an imbalance of neurotransmitters in the basal ganglia circuitry pathways (Calabresi et al., 2014). Another pathological feature of PD is the intracellular aggregation of α-synuclein proteins, or also known as the formation of Lewy bodies (LBs), which is said to originate from the olfactory bulb and medulla oblongata then to other brain regions as the disease progresses (Stefanis, 2012; Barnhill et al., 2020). The formation of LBs impairs the ubiquitin-proteasome protein degradation process and consequently heightens the number of α-synuclein aggregates (Bentea et al., 2017). Besides that, PD patients were reported to have a deficient level of mitochondrial complex I, an enzyme especially responsible for the mitochondrial electron transport chain (Mimaki et al., 2012). An insufficient level of this enzyme leads to failure in energy production (in the form of ATP), which eventually contributes to mitochondrial dysfunction (Kouli et al., 2018). Apart from providing energy to the neurons, mitochondria also act as a major calcium ion storage, however, perturbed mitochondrial physiology impairs calcium ion regulation (Zaichick et al., 2017). A high level of calcium ions in the cytosol is toxic to the neurons, impairs the autophagy process, and is even said to stimulate the aggregation of α-synuclein (Michel et al., 2016). As a result, the accumulation of α-synuclein aggregation causes neuronal toxicity and increases neuronal susceptibility to programmed cell death (Bentea et al., 2017; Surmeier, 2018).

### PD Pathogenesis and Neurotransmitter Imbalances

Progressive neurodegeneration of PD results in neurotransmitter imbalances and alters the neurotransmission systems. It has been well documented that the most affected neurotransmitter in PD pathogenesis is dopamine (Barone, 2010). Dopamine neurotransmitter is a type of catecholamine which normally acts as an excitatory or inhibitory neurotransmitter, depending on the type of receptor that it binds to Sayin (2019). The dopamine neurotransmission is involved in many cognitive and emotional processes, including attention, memory, and reward processing (Bromberg-Martin et al., 2010; Klein et al., 2019). In PD pathophysiology, the dopaminergic neuronal death in the SNpc leads to a substantial reduction of striatal dopamine population and subsequent decrease in dopamine projection to other brain regions (Delaville et al., 2011). Furthermore, dopamine production in the SNpc is modulated by other neurotransmitters, namely glutamate and GABA (Barone, 2010). Since dopamine is affected by PD pathology, this phenomenon causes neurotransmission imbalance and altered the communication between dopamine, glutamate, and GABA neurotransmitters. A study on a rodent model of PD reported an increase in glutamate concentration in the tail of the ventral tegmental area following a unilateral 6-OHDA lesion in the SNpc (Chang et al., 2019). This suggests that dopamine deficiency alters the equilibrium of other neurotransmitters not just in the SNpc, but in other related regions as well.

Other biogenic amines such as norepinephrine, serotonin, and histamine are also highly impacted in PD (Sawada et al., 2013; Jamwal and Kumar, 2018). Reduced dopaminergic neuronal function in PD is reported to increase norepinephrine activity, possibly a feedback mechanism by norepinephrine as an excitatory drive to increase the dopamine back to normal level (Delaville et al., 2011; Werner and Coveñas, 2017). However, it is striking to note that the locus coeruleus is also affected by PD pathology, hence the synthesis of norepinephrine is significantly reduced as the disease progresses. Norepinephrine reduction further damages dopamine production and increases the susceptibility of DA neurons to degenerate (Barone, 2010; LeWitt, 2012). Similarly, it is reported that serotonin or 5-hydroxytryptamine (5-HT) concentration is reduced in PD and may associate with the presentation of postural tremor in PD patients (Pasquini et al., 2018). Other than tremor, reduction in serotonin is also associated with non-motor symptoms of PD, such as depression, fatigue, and visual hallucinations (Politis and Loane, 2011). Concerning histamine, the brain of PD patients recorded an enhanced activity of histamine in the SNpc and globus pallidus (Bolam and Ellender, 2016). Increased activation of histamine in these regions is toxic to the surrounding neurons as it will release ROS that can damage the neurons due to oxidative stress (Rocha et al., 2014). In normal physiology, all neurotransmitters work in concert with each other, however, in PD, neurotransmitter imbalances take place which leads to the dysfunction of the basal ganglia circuitry.

## Zebrafish as an Animal Model

### Historical Background

The involvement of zebrafish in the research world is said to begin in the 1950s by a molecular biologist, George Streisinger, from the University of Oregon. Due to the transparent nature of zebrafish larvae, Streisinger believed that it is possible to study the neural development of the vertebrate, a knowledge that was limited at that time due to less developed techniques and unsuitable animal models (Barros et al., 2008; Eisen, 2020). Valuable findings from Streisinger and his colleagues regarding neural development have captured the interest of many other researchers to use zebrafish in their research as well. Ten years since the introduction of this species, researchers have managed to perform a plethora of investigations on zebrafish and gain deeper knowledge, especially on those related to the development of the nervous system (Rahman Khan and Sulaiman Alhewairini, 2019; Eisen, 2020). The study on zebrafish kept expanding and by the 1900s, standardized techniques on zebrafish husbandry were developed (Meyers, 2018) and genetic information was made transparent in online databases and repositories (Holtzman et al., 2016). In 1998, the National Institute of Health (NIH) established the first-ever zebrafish initiative known as the Trans-NIH Zebrafish Initiative (Freeman et al., 2007; Eisen, 2020), which documented the acceptance of zebrafish as an animal model in scientific studies. Since then, zebrafish have been used as a model organism in various research areas including neurodegeneration, neurodevelopment, neurobehavior, toxicology, and drug discoveries (D’Amora and Giordani, 2018; Saleem and Kannan, 2018).

Similar to other species of animal models, several different zebrafish strains are being used in biomedical research. According to the ZFIN (The Zebrafish Information Network) database, more than 10 wild-type zebrafish strains are available, with the AB, TU (Tübingen shortfin), and WIK (wild Indian karyotype) being the most widely studied and well-documented strains (Audira et al., 2020). In a study of the variances between several zebrafish strains (wild-type and laboratory strains), it is revealed that since the strains came from different origins (for example, AB is from Oregon, United States while TU is from Tübingen, Germany, and WIK is from Bangladesh, India), there are some distinct genetic and phenotypic variances between the strains, suggesting the phenomena of genetic drifts and bottlenecks (Suurväli et al., 2020). The WIK strain is found to be more anxious and less aggressive compared to the AB and TU strains, which is reflected by increased thigmotaxis behavior (Kalueff et al., 2014). AB strain, on the other hand, expresses a higher level of stress hormone that results in increased aggressiveness than TU and WIK strains (Manuel et al., 2016). Hence, it is important to be reminded that strain-specific behaviors should be considered when interpreting zebrafish behaviors in a study (Audira et al., 2020). Nevertheless, DNA sequencing revealed that the AB and TU strains are genetically related to each other, thus possessing a high chance of result reproducibility (Suurväli et al., 2020). Not including the transgenic lines, other strains of wild-type zebrafish include EKW, Nadia, and Casper (see Kalueff et al. for further explanation). Taken together, in choosing the best strain to be used for a study, it depends on the aim and the experimental design of that particular study. It is interesting to note that, in terms of within-strain variances, the AB strain is reported to have the lowest coefficient of variation (Audira et al., 2020), an advantage that should be considered in choosing an animal model for research.

### Zebrafish Model in Behavioral Neuroscience

**FIGURE 1:**
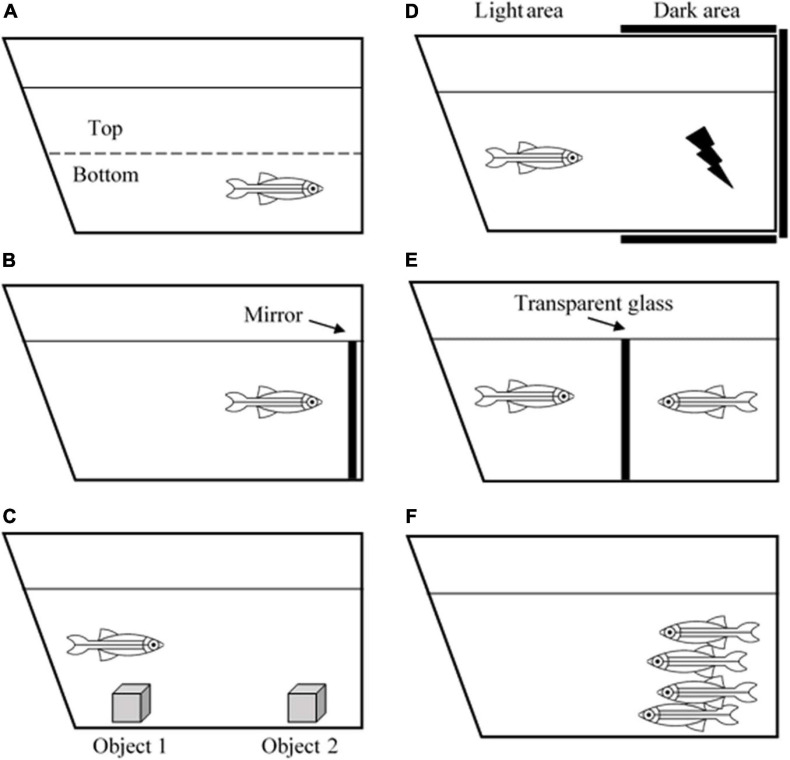
Illustration of zebrafish behavioral tests. **(A)** Novel tank test, **(B)** mirror test, **(C)** object discrimination test, **(D)** avoidance learning test, **(E)** male-male interaction test, and **(F)** shoaling test.

Zebrafish is an ideal model of behavioral neuroscience because like humans, zebrafish also possess arrays of cognitive processes including learning, memory, fear, anxiety, perception, social skills, and even sleep pattern (Oliveira, 2013). Currently, several well-developed tests and paradigms are becoming increasingly available to study zebrafish behaviors (Costa et al., 2020). A novel tank task and mirror test are used to measure anxiety (Figure 1). Zebrafish that exhibit high anxiety levels spend more time at the edge of a novel tank than at the center (thigmotaxis) and the bottom of the tank than on the top (Haghani et al., 2019). Aberrant swimming behaviors, such as increased freezing bouts and reduced inter-distance between fishes in a shoal also correspond to the anxiety level (Speedie and Gerlai, 2008; Blaser et al., 2010). Interestingly, due to the high basal level of cortisol hormone in the brain, the AB wild-type strain has been the most ideal zebrafish strain for anxiety and aggression studies (Oliveira, 2013).

In terms of learning and memory functions, zebrafish can process associative learning, avoidance learning, object discrimination learning, spatial learning, and several more (Norton and Bally-Cuif, 2010). For example, the object discrimination test measures the ability of a zebrafish to retain a memory (Figure 1). Zebrafish are introduced to a novel object to test whether they are still able to recognize the object after 1- or 24-h post-introduction (Stefanello et al., 2019). One study revealed that the zebrafish telencephalon and thalamus are the major brain regions that are involved in processing visual discrimination (Messina et al., 2020). Another behavioral task, aiming to evaluate avoidance learning and memory, is the avoidance learning test that requires the zebrafish to learn to avoid an electric shock by refraining to swim into a dark compartment (Figure 1). The task reveals that zebrafish can acquire avoidance learning and process it into long-term memory (Blank et al., 2009).

Apart from that, zebrafish are found to be a social species. They exhibit signs of aggressiveness, anxiety, shoal mate preference, and kin recognition. Such tests that can be used to measure these behaviors include the mirror test, male-male interaction test, and shoaling test (Figure 1; Audira et al., 2018). Besides, since they live in groups or shoals, they also have hierarchical dominance and territoriality (Spence, 2011). These behaviors are especially beneficial for the behavioral research world because humans are a highly social species, and they depend on social cooperation to thrive and survive. Apart from social skills, zebrafish are also utilized for sleep studies. It is reported that zebrafish possess a circadian rhythm that regulates their sleep-wake cycle similar to humans. Leung et al. (2019) revealed that zebrafish have two major sleep patterns known as the slow bursting sleep and propagating wave sleep patterns, which are equivalent to human slow-wave sleep and rapid eye movement sleep, respectively. All of the examples have given an adequate explanation on the use of zebrafish as an animal model for neuro-behavioral studies. Their social plasticity and the ease of manipulating genotypic and phenotypic characteristics make this species an excellent model to study the correlation between a perceived behavior and what is happening inside the brain.

### Advantages and Challenges of Using Zebrafish as an Animal Model

#### Advantages of the Zebrafish Model

Zebrafish are vertebrates, hence, comparing to other non-mammalian models like *C. elegans* and *D. melanogaster*, they are anatomically closer to humans. While there are some significant variations between the brain of the zebrafish and that of humans in terms of forms and sizes, the overall organization shows similarity. When compared with their human counterparts, unique areas of the zebrafish brain may be linked to and are often strikingly preserved. For instance, the zebrafish ventral telencephalon is proposed to be homologous to the human striatum. Besides, the behaviors and phenotypes demonstrated by zebrafish are relatable to human behaviors. For example, neurotoxin-induced zebrafish exhibits movement impairments such as decreased swimming speed and abnormal swimming behavior, which is equally translated to bradykinesia-like symptoms in PD patients (Hughes et al., 2020; Robea et al., 2020). In terms of genetic factors, genome sequencing analysis revealed that the zebrafish genome is 70% similar to the human genome (Howe et al., 2013; Shehwana and Konu, 2019), with 80% of the genes located at the same chromosome and in the same order, implying conserved synteny between these two species (Barbazuk et al., 2000; Howe et al., 2013). The genetics of the zebrafish model are well-characterized, and the transgenic models are well-documented (Kalueff and Cachat, 2011). Furthermore, what makes zebrafish an extremely special model is that its embryos develop externally and are transparent, hence, the developmental process is possible to be studied in real-time (Ali et al., 2011; Rahman Khan and Sulaiman Alhewairini, 2019) and the embryos can readily absorb given compounds or neurotoxins (D’Amora and Giordani, 2018).

Other advantages of zebrafish over other animal models include high fecundity and short life cycle. Zebrafish can lay as much as 200–300 eggs per week (Hoo et al., 2016; Rahman Khan and Sulaiman Alhewairini, 2019). This gives an excellent advantage to this species because a higher sample size can be applied to each experiment and more significant results can be achieved. Besides that, zebrafish only need 3–4 months to achieve sexual maturity (Njiwa et al., 2004) and its average life span is only up to 3–4 years (Gilbert et al., 2013). The short life cycle can substantially reduce the time taken and lower the cost of an experiment, especially when the experimental design requires investigation on a full developmental process. Moreover, the protocols for husbandry and maintenance of zebrafish are much easier and less complicated to handle compared to rodents and non-human primates (Avdesh et al., 2012).

#### Challenges of the Zebrafish Model

It cannot be denied that zebrafish have provided various advantages that have led to valuable observations and new findings. However, several shortcomings should be noted when choosing this species as the model of a study. Firstly, accessible information regarding zebrafish strains and transgenic models is still much less developed compared to other model organisms like rodents (Goldsmith and Jobin, 2012; Stewart et al., 2014). Besides, only a small number of validated reagents (such as test kits and antibodies) that react with zebrafish are available, hence limiting the post-sacrifice molecular analyses (Goldsmith and Jobin, 2012).

Next, in terms of evaluating drug efficacy, although the external development of zebrafish embryos allows real-time observation, however, the uptake of the drug by each embryo may vary, especially when administered orally, thus leading to heterogeneity of results (Lardelli, 2014). Also, since the embryos develop externally, the metabolism pathway and rate of uptake of certain drugs or chemicals may be different from that of human embryos (Ali et al., 2011; Saleem and Kannan, 2018). Hence, detailed evaluation must be made to determine the effect dose to avoid result misinterpretations.

Apart from that, zebrafish possess the natural ability to perform regeneration and neurogenesis for the replacement of cell injury or cell death(Gemberling et al., 2013; Barbosa and Ninkovic, 2016). For example, the dopaminergic neuronal population in the olfactory bulb of adult zebrafish was regenerated to the normal level within 45 days of post-ablation (Godoy et al., 2020). This ability has brought some difficulties to PD studies because it makes the establishment of a PD model more challenging. Although disadvantageous in the context of degenerative studies, nevertheless, knowledge on zebrafish regenerative ability is beneficial as it provides a promising platform for studying regenerative pathways and possibly applying it to humans (Gemberling et al., 2013; Marques et al., 2019).

Lastly, while humans have two MAO subtypes (MAO-A and MAO-B), zebrafish have only one type of MAO, termed as the zMAO (Arslan and Edmondson, 2010). Although no known tertiary protein structure is currently available in the literature, a hypothetical substrate-binding site indicates that the zMAO is functionally more similar to human MAO-A than MAO-B (Arslan and Edmondson, 2010; Fierro et al., 2013). Since MAO is very essential as it breaks down important neurotransmitters in the brain and is said to participate in PD pathophysiology, zebrafish studies involving the mechanism of action of this enzyme should be cautiously interpreted. Table 1 below summarizes the advantages and challenges of using zebrafish as an animal model.

**TABLE 1:** Summary of the advantages and challenges of using zebrafish as a model organism.

**Advantages**	**References**
– Vertebrate animal—anatomically closer to human than other invertebrate models – Behaviors relatable to human – Close genome homology to human – Well-characterized genetics and transgenic models – Embryos are transparent and develop externally (outside mother)—real-time observation. – High fecundity – Short life cycle – Less complicated husbandry and maintenance	Barbazuk et al., 2000; Njiwa et al., 2004; Ali et al., 2011; Kalueff and Cachat, 2011; Avdesh et al., 2012; Gilbert et al., 2013; Howe et al., 2013; Hoo et al., 2016; D’Amora and Giordani, 2018; Rahman Khan and Sulaiman Alhewairini, 2019; Shehwana and Konu, 2019; Hughes et al., 2020; Robea et al., 2020

**Challenges**	**References**

– Less developed strains and transgenic models – Limited availability of molecular reagents and kits – Variable compound uptake for embryos – Different metabolism pathway and rate of compound uptake (since human embryos develop internally) – Neurogenesis ability—opposes degeneration process. – Functional difference of MAO	Arslan and Edmondson, 2010; Ali et al., 2011; Goldsmith and Jobin, 2012; Fierro et al., 2013; Gemberling et al., 2013; Lardelli, 2014; Stewart et al., 2014; Barbosa and Ninkovic, 2016; Saleem and Kannan, 2018; Marques et al., 2019; Godoy et al., 2020

## Zebrafish as a Model of Parkinson’s Disease

It is a well-known fact that anatomically, zebrafish relate closer to humans compared to its invertebrate counterparts, *C. elegans* and *D. melanogaster*. Not just that, between zebrafish and humans, relies similar quantifiable behaviors and pathophysiology. From its first official announcement as a model organism in 1998 until recently, extensive studies involving zebrafish have led to the mapping of its dopaminergic nervous system (Tay et al., 2011). Being extensively studied on the nervous system, zebrafish made a suitable candidate for the study of PD. The behaviors exhibited by zebrafish are similar and easily translated to those shown by humans (Best and Alderton, 2008), hence, PD studies using zebrafish are not only limited to genotypic and molecular mechanisms, but they also provide information on phenotypic factors.

The earliest presence of DA neurons in zebrafish embryo was detected upon its 19th hour of post-fertilization (hpf) in the posterior tuberculum of ventral diencephalon–the region that is equivalent to human SN, and the cluster of these neurons was shown to project to ventral telencephalon–zebrafish equivalence to human striatum. The whole process is believed to represent the nigrostriatal dopaminergic nervous system (Flinn et al., 2008), which is a feature that is undoubtedly important in PD pathology. Substantial information on DA neuronal projections in zebrafish brain and its compatibility in experimental manipulations have given this species a clear advantage in studying molecular mechanisms related to PD.

Typically, there are two types of zebrafish model of PD: neurotoxin-induced model and transgenic model (Xi et al., 2011). MPTP is the most common neurotoxin used to induce PD in zebrafish. It triggers the degeneration of DA neurons and reduces the levels of dopamine, norepinephrine, and serotonin in the brain, specifically in the posterior tuberculum of the ventral diencephalon (Vaz et al., 2018). Zebrafish PD model induced with MPTP exhibits motor dysfunctions such as reduced swimming speed and abnormal swimming behaviors, which are translated to bradykinesia in PD patients (Hughes et al., 2020). Another widely used neurotoxin is 6-hydroxydopamine (6-OHDA), which is an oxidative dopamine analog that causes mitochondrial dysfunctions that leads to DA and noradrenergic neuronal death (Zeng et al., 2018). The transgenic model, on the other hand, uses transgenic type zebrafish that expresses targeted mutated genes to mimic autosomal dominant or recessive PD in humans (Xi et al., 2011).

### Neurotoxin-Induced Zebrafish Model of PD

As mentioned earlier, there are several available neurotoxins such as MPTP, 6-OHDA, paraquat, and rotenone that have been used throughout decades in zebrafish studies to induce PD-like symptoms. Subsections below discuss the mechanism of action of each neurotoxin and its neurobehavioral effects on the zebrafish model of PD.

#### 1-methyl-4-phenyl-1,2,3,6-tetrahydropyridine (MPTP)

1-methyl-4-phenyl-1,2,3,6-tetrahydropyridine (MPTP) was discovered in the early 1980s through drug users who developed parkinsonism upon injecting themselves with a meperidine analog, the 1-methyl-4-phenyl-4-propionoxypiperidine (MPPP) (Stepens and Taba, 2016; Langston, 2017). MPTP is a by-product of MPPP (Langston, 2017). Later, it was found that besides humans, MPTP can trigger parkinsonism in other animal species like primates, rodents, and zebrafish as well (Vaz et al., 2018). The mechanism of action of MPTP toxicity in zebrafish is similar to humans (Barnhill et al., 2020), hence its extensive use as a PD model is justified. The discovery of MPTP has opened a new promising opportunity in PD studies because this compound can easily cross the blood brain barrier (BBB) (Zeng et al., 2018). MPTP is metabolized in glial cells by monoamine oxidase B (MAO-B), which causes the conversion of MPTP to its active metabolite, MPP + (Bajpai et al., 2013; Robea et al., 2020). Due to its similar structure with dopamine, MPP + is then transported into DA neurons by dopamine transporter protein (DAT) (Fernagut, 2016). Inside the neurons, MPP + is concentrated inside the mitochondria where it blocks mitochondrial electron transport chain through binding with mitochondrial complex I (Zawada et al., 2011). The binding of MPP + with complex I perturb the subsequent electron transport chain, leading to insufficient energy generation (in the form of ATP) and increased ROS production (Perier and Vila, 2012; Robea et al., 2020).

Upon exposure to MPTP, zebrafish exhibit movement impairments, such as reduced swimming speed and aberrant swimming behavior, which are translated to bradykinesia-like symptoms in humans. Besides that, zebrafish spend longer time at the bottom of the tank and the number of freezing bouts increases, showing a significant reduction in locomotor activities. Moreover, MPTP is also reported to weaken the touch sensory. Zebrafish exposed to MPTP responded poorly to tactile stimuli, manifested by slow trunk and tail reflexes in response to the stimuli (Lam et al., 2005; Wasel and Freeman, 2020). The sensory deficit is possibly caused by a reduction in the striatal dopamine population that impairs striatal functioning (Ketzef et al., 2017, a rodent study).

#### 6-hydroxydopamine (6-OHDA)

6-hydroxydopamine (6-OHDA) is another type of neurotoxin that has been routinely used to induce PD-like symptoms in animal models. However, unlike the MPTP, 6-OHDA is not permeable to the BBB, hence the administration of 6-OHDA is usually done by injecting the neurotoxin directly into the targeted brain region (Hernandez-Baltazar et al., 2017). This neurotoxin is a highly oxidizable dopamine analog that is taken up by DAT into DA neurons (Vijayanathan et al., 2017). Upon entering the neurons, monoamine oxidase (MAO) oxidizes 6-OHDA to quinone and by-products such as hydrogen peroxide and other free radical molecules (Cobley et al., 2018). The production of ROS by-products elicits oxidative stress-related toxicity and microglia-mediated neuroinflammation at the targeted region (Vijayanathan et al., 2017). Apart from generating the ROS, 6-OHDA is also reported to directly inhibit the activity of mitochondrial complex I, thus causing mitochondrial dysfunction (Kesh et al., 2021). Overall, the oxidative stress and the inhibition of complex I by 6-OHDA trigger the progressive loss of DA neurons and damages the nigrostriatal DA pathway.

The induction of 6-OHDA on zebrafish stimulates the death of DA neurons and subsequent reduction of dopamine levels in the lesioned area (Vaz et al., 2020). Vijayanathan et al. (2017) reported a substantial loss of DA neurons within 12 h after 6-OHDA administration. In terms of the behavioral effects, the same study observed significant reductions in swimming speed and distance in 6-OHDA-induced zebrafish (Vijayanathan et al., 2017). Slower speed and shorter swimming distances are similar to akinesia seen in PD patients (Makhija and Jagtap, 2014). In another study, 6-OHDA exposure to larval zebrafish reduced burst swimming (a swimming behavior for zebrafish larvae) 2 days after the exposure (Vaz et al., 2020). Plus, similar to the adult zebrafish, larval zebrafish also exhibit shorter swimming distances when exposed to 6-OHDA (Feng et al., 2014). Additionally, 6-OHDA also stimulates anxiogenic behavior, which is a common non-motor symptom of PD (Carvalho et al., 2013; Bonito-Oliva et al., 2014). Larval zebrafish group treated with 6-OHDA spent a longer time at the lower part of the cuvette compared to the normal group, indicating a sign of anxiety (Feng et al., 2014). Hence, on the spectrum of behavioral effects, 6-OHDA administration impairs the locomotor activity and induces anxiety level of the zebrafish model of PD.

#### Paraquat

Paraquat (1-1′-dimethyl-4,4′-bipyridine) is a weedkiller herbicide that has a structural similarity with the MPTP neurotoxin (Vaccari et al., 2019). It was first discovered in 1985 in a frog study that paraquat triggers dopamine reduction and behavioral changes, similar to the effects seen in MPTP-induced models (Barbeau et al., 1985). Although both MPTP and paraquat prompt similar physiological effects, their mechanism of action is different, because unlike the lipophilic MPTP, paraquat is hydrophilic and thus, unable to passively cross the BBB (Tangamornsuksan et al., 2019; Vaccari et al., 2019). In this regard, the translocation of paraquat from outside to inside of the CNS might occur via an uncharged amino acid transporter (Tangamornsuksan et al., 2019). Inside the CNS, paraquat in the form of di-cation crosses the membrane of inner mitochondria and is further reduced to mono-cation. Paraquat radical mono-cation is highly reactive with oxygen, leading to the propagation of ROS (Nunes et al., 2017). Indeed, the most prominent cause of paraquat-induced neurotoxicity is the elevated production of superoxide and hydrogen peroxide (Wang et al., 2018). Additionally, though paraquat toxicity triggers mitochondrial dysfunction, but unlike MPTP, paraquat is less likely to inhibit the mitochondrial complex I activity (Joseph et al., 2020).

*In vivo* PD studies using the zebrafish model indicate that paraquat impairs mitochondrial function and dopaminergic signaling (Wang et al., 2018; Vaccari et al., 2019). Redox imbalance caused by the increased mitochondrial ROS production causes oxidative stress and damages mitochondrial respiration (Bastías-Candia et al., 2019). Supporting this, Joseph et al. (2020) reported that paraquat exposure to zebrafish larvae reduced mitochondrial viability and obstructed the production of ATP. Interestingly, paraquat is also found to activate nuclear factor-kappa B (NFκB) that results in DNA fragmentation and TLR4/NFκB-mediated neuroinflammation (Feng et al., 2019; Huang et al., 2020). Behavioral-wise, paraquat reduces locomotor activity of zebrafish, indicated by decreased swimming distance and velocity (Wang et al., 2018). This finding agrees with Joseph et al. (2020), who documented an increase in the number of freezing bouts and freezing durations of zebrafish after paraquat administration. Moreover, the same study also found that paraquat impairs the process of acquiring and consolidating spatial memory. Regarding the non-motor symptoms of PD, paraquat elicits anxiogenic and aggressive behaviors of adult zebrafish, as observed in the open field test and mirror test, respectively (Nunes et al., 2017).

#### Rotenone

Rotenone is a complex ketone derived naturally from lancepod, which is a leguminous plant and is used as an herbicide, insecticide, and piscicide (Bastías-Candia et al., 2019). Since rotenone is a natural compound, it can be degraded by air, water, and light within a few days (Robea et al., 2018). However, in 1985, rotenone was discovered to induce DA neuronal toxicity by triggering mitochondrial dysfunction and oxidative stress (Bastías-Candia et al., 2019). Rotenone easily crosses the BBB and enters the CNS due to its lipophilic structure (Robea et al., 2018; Bastías-Candia et al., 2019). Unlike paraquat that needs amino acid transporter to enter the CNS and MPP + that needs DAT to enter the DA neurons, rotenone directly enters the neurons and accumulates in cellular organelles, predominantly in the mitochondria (Ünal et al., 2020). The rotenone mechanism of action involves inhibiting mitochondrial complex I activity, leading to the reduction of ATP production and an increase in ROS (Yurtsever et al., 2020). Reportedly, rotenone also triggers microglial activation which results in microglial-induced neuroinflammation (Gao et al., 2013) and stimulates α-synuclein aggregation which leads to the Lewy body pathology (Hijaz and Volpicelli-Daley, 2020).

In zebrafish PD studies, rotenone has been reported to reduce the dopamine population, marked by a decreased level of tyrosine hydroxylase expression (Wang et al., 2015; Lv et al., 2019; Ramli et al., 2020). Furthermore, rotenone was observed to reduce mitochondrial calcium levels (Yurtsever et al., 2020). Mitochondria acts as a major calcium ion storage and a disturbance to the mitochondrial calcium homeostasis is associated with PD pathogenesis (Scorziello et al., 2020). Also, Yurtsever et al. (2020) documented that rotenone-treated zebrafish expressed an increased level of pro-inflammatory proteins, such as tumor necrosis factor α (TNFα), interleukin-21 (IL-21), and NFκB. In the behavioral context, rotenone elicits motor dysfunctions on zebrafish that are consistent with the motor features of PD (Wang et al., 2015). Adult zebrafish treated with rotenone swum slower and traveled shorter distances compared to the non-treated group (Ünal et al., 2019; Ramli et al., 2020). Indeed, a slower swimming ability is reflective of the bradykinesia-like symptom of PD (Lv et al., 2019). Apart from that, rotenone is also reported to induce several non-motor symptoms on zebrafish. Wang et al. (2015) showed that 4 weeks of rotenone treatment in zebrafish triggered anxiety and depression, as measured through the light-dark box test. Furthermore, the same study also reported impaired olfaction in rotenone-treated zebrafish, which is another common non-motor symptom of PD. Table 2 below summarizes the observed physiological and behavioral effects of the environmental neurotoxins on the zebrafish model of PD.

**TABLE 2:** Summary of the physiological and behavioral effects observed on the neurotoxin-induced and transgenic zebrafish model of PD.

**Environmental neurotoxin**	**Observed effects upon exposure on zebrafish**		**References**
	**Physiological effects**	**Behavioral effects**	
**Neurotoxin-induced zebrafish model of PD**			
MPTP	– Perturbation of mitochondrial electron transport chain through binding with mitochondrial complex I – Reduced ATP production – Increased ROS production	– Motor impairments (reduced swimming speed, aberrant swimming behavior, increased time spent at the bottom of the tank, increased number of freezing bouts) – Weakened touch sensory (slow trunk and tail reflexes in response to the stimuli)	Lam et al., 2005; Zawada et al., 2011; Perier and Vila, 2012; Ketzef et al., 2017; Robea et al., 2020; Wasel and Freeman, 2020
6-OHDA	– Inhibition of the mitochondrial complex I activity – Increased ROS production – Reduced dopamine level – Activation of microglia-mediated neuroinflammation	– Motor impairments [reduced swimming speed, reduced distance traveled, reduced burst swimming (larval zebrafish)] – Anxiety (longer time spent at the lower part of the cuvette)	Carvalho et al., 2013; Bonito-Oliva et al., 2014; Feng et al., 2014; Makhija and Jagtap, 2014; Vijayanathan et al., 2017; Cobley et al., 2018; Vaz et al., 2020; Kesh et al., 2021
Paraquat	– Increased ROS production – Reduced ATP production – Activation of the TLR4/NFκB neuroinflammatory pathway – DNA fragmentation	– Motor impairments (reduced distance traveled, reduced swimming velocity, increased number of freezing bouts and freezing durations) – Impaired spatial memory – Anxiety – Aggression	Nunes et al., 2017; Wang et al., 2018; Bastías-Candia et al., 2019; Feng et al., 2019; Vaccari et al., 2019; Huang et al., 2020; Joseph et al., 2020
Rotenone	– Inhibition of the mitochondrial complex I activity – Increased ROS production – Reduced ATP production – α-synuclein aggregation – Reduced dopamine level – Reduced mitochondrial calcium level – Increased activation of microglia and pro-inflammatory proteins	– Motor impairments (reduced swimming speed, reduced distance traveled) – Anxiety – Depression – Olfactory dysfunction	Gao et al., 2013; Wang et al., 2017; Lv et al., 2019; Hijaz and Volpicelli-Daley, 2020; Ramli et al., 2020; Ünal et al., 2020; Yurtsever et al., 2020

**PD-associated gene/Encoded protein**	**Observed effects of mutations on zebrafish**		**References**

	**Physiological effects**	**Behavioral effects**	
**Transgenic zebrafish model of PD**			
*SNCA/*Synuclein	– Reduced dopamine level – Reduced mitochondrial activity – Increased ROS production	– Motor impairment (reduced spontaneous swimming behavior)	Milanese et al., 2012; O’Donnell et al., 2014; Grünewald et al., 2019; Robea et al., 2020
*PARK2*/Parkin	– Reduced mitochondrial activity – Reduced DA neuronal population – Increased susceptibility to toxic metabolites	– No significant swimming behavioral abnormality	Flinn et al., 2009; Vaz et al., 2018; Wasel and Freeman, 2020
*PINK1/*PINK1	– Mitochondrial dysfunction – Developmental retardation – Increased ROS production – Increased susceptibility to MPTP	– Motor impairment (abnormal swimming behavior) – Impaired response to tactile stimuli	Anichtchik et al., 2008; Sallinen et al., 2010; Xi et al., 2010; Priyadarshini et al., 2013
*PARL/*PARL	– Dysregulation of the PINK1/Parkin mitophagy pathway – Reduced DA neuronal population – Increased mortality	– Motor impairment (reduced distance traveled and swimming velocity, increased freezing bouts) – Olfactory dysfunction	Noble et al., 2012; Shamchuk et al., 2017; Merhi et al., 2021
*PARK7/*DJ-1	– Increased ROS production – Impaired mitophagy	– Motor impairment (reduced swimming velocity, increased freezing bouts)	Dolgacheva et al., 2019; Edson et al., 2019
*LRRK2/*LRRK2	– Neuronal cell loss – Synuclein aggregation – Heightened kinase activity – Weakened immunity toward bacterial infection	– Motor impairment (reduced swimming distance)	Sheng et al., 2010; Prabhudesai et al., 2016; Sheng et al., 2018; Seegobin et al., 2020

The zebrafish study of PD using environmental neurotoxins has provided valuable information on the pathophysiological mechanism of this disease. Despite its usefulness and advantages, several limitations need to be pointed out when using the neurotoxin-induced zebrafish model. For instance, the effects exerted by the neurotoxins are acute and transient (Duty and Jenner, 2011; Palasz et al., 2019). The effect of MPTP on zebrafish reached its maximum level at 48 h post-administration and started to wear-off on day 12 (Anichtchik et al., 2004). By contrast, PD is a progressive disease that is prolonged and gradually worsens over time. Considering this limitation, it is challenging to relate the acute exposure to neurotoxin to the different stages of PD progression. Furthermore, some of the neurotoxins like the MPTP and 6-OHDA are unable to induce the α-synuclein aggregation to the zebrafish, hence they do not completely recapitulate the pathophysiology of PD in humans (Zeng et al., 2018). Since the α-synuclein aggregation is considered as the main etiological factor in PD pathogenesis, and an acute exposure to neurotoxin cannot trigger the aggregation, attention is being given to the transgenic model of zebrafish (Chia et al., 2020). In some *in vivo* studies, the human α-synuclein protein is introduced with or without neurotoxins to the wild-type zebrafish to have a better representation of the PD pathogenesis (O’Donnell et al., 2014; Weston et al., 2021). More detailed discussions on the transgenic zebrafish model are discussed in the next section.

### Transgenic Zebrafish Model of PD

Decades of extensive studies have led to the identification of several genes associated with PD development in zebrafish. These genes include the *SNCA, PARK2, PINK1, PARL, PARK7*, and *LRRK2* genes (Nuytemans et al., 2010; Klein and Westenberger, 2012), that encode for synuclein, Parkin, PINK1, PARL, DJ-1, and LRRK2 proteins, respectively, all of which have relatively close homology and similar functions to those expressed in human.

#### α-Synuclein (*SNCA*) Gene

The *SNCA* gene in humans encodes for α-synuclein protein. Although the exact function of this protein in normal conditions is still unclear, it is believed to be responsible for regulating the synaptic transmission process (Marques and Outeiro, 2012; Burré, 2015). However, in a pathological condition, misfolded α-synuclein proteins form aggregates, which eventually leads to the formation of Lewy bodies (LBs) (Mahul-Mellier et al., 2020). Aggregation of LBs is the hallmark of PD pathology (Kouli et al., 2018) and mutations in the SNCA gene facilitate the development of early onset PD (Bridi and Hirth, 2018).

Zebrafish lacks the expression of α-synuclein, nevertheless, they do express three synuclein genes known as the *SNCB, SNCG1*, and *SNCG2*, that encode for β-, γ 1-, and γ2-synuclein proteins, respectively (Toni and Cioni, 2015). Recent investigation suggested that the γ1-synuclein is functionally similar to human α-synuclein (Barnhill et al., 2020). The knockdown of *SNCB* and *SNCG1* genes in zebrafish caused a significant reduction in motor activity and lowered the level of dopamine in the brain (Milanese et al., 2012), indicating that zebrafish β- and γ1-synuclein proteins are required for movement regulation and dopamine homeostasis (Toni and Cioni, 2015; Vaz et al., 2018; Robea et al., 2020).

The advancement in genetic technology has enabled the construction of zebrafish transgenic models that express human wild-type α-synuclein protein (Prabhudesai et al., 2012; O’Donnell et al., 2014; Van Laar et al., 2020; Weston et al., 2021). Over-expression and aggregation of α-synuclein protein in zebrafish model of PD exhibited reduced mitochondrial activity and increased presence of reactive oxygen species (ROS), which led to neuronal apoptosis and cell death (O’Donnell et al., 2014; Robea et al., 2020). Evidence from recent studies emphasized that intracellular LBs progressively perturb the population of DA neurons, possibly by affecting mitochondrial functions and inducing oxidative stress to the neurons, thus leading to early onset PD (Grünewald et al., 2019).

#### Parkinson’s Disease Protein 2 (*PARK2*) Gene

Mutations in the *PARK2* gene are associated with early onset PD (Selvaraj and Piramanayagam, 2019). This gene is translated to Parkin protein, and mutations of this gene are the most common cause of autosomal recessive PD (Selvaraj and Piramanayagam, 2019; Barnhill et al., 2020). In normal conditions, Parkin functions as a ligase that targets other proteins for degradation. Specifically, Parkin identifies damaged proteins due to cell insult or injury and performs autophagy and breakdown processes to clear out the injured proteins (Barnhill et al., 2020). Parkin is also responsible for mitophagy, a process of degrading damaged mitochondria, generally through PINK1/Parkin pathway (Ashrafi and Schwarz, 2013). Furthermore, Parkin targets and degrades α-synuclein proteins (Khandelwal and Moussa, 2010). In a pathological condition, mutated *PARK2* gene encodes for loss-in-function Parkin protein, which disabled its degrading ability, thus increasing the risk of developing PD through mitochondrial dysfunction, α-synuclein aggregation, and LB formation (Selvaraj and Piramanayagam, 2019; Ge et al., 2020). Nonetheless, the association between *PARK2* mutation and LB formation is believed to be incidental or secondary (Doherty and Hardy, 2013; Madsen et al., 2021).

The zebrafish Parkin protein is 62% homologous and 78% functionally similar to human Parkin (Barnhill et al., 2020). Knockdown of *PARK2* gene in zebrafish using morpholino oligonucleotides significantly reduced mitochondrial complex I activity, thus utterly disrupting the mitochondrial respiratory chain (Wasel and Freeman, 2020). Supporting this, the zebrafish model of PD with a knockdown *PARK2* gene showed a 20% reduction in the number of DA neurons in the diencephalon, but no abnormalities in swimming behavior was detected (Flinn et al., 2009). Besides that, Parkin-deficient zebrafish is more sensitive to the toxic metabolite of MPTP, 1-methyl-4-phenylpyridinium ion (MPP+), compared to the wild type (Flinn et al., 2009; Vaz et al., 2018). Hence, the inability of Parkin protein to regulate mitochondrial condition is suggested to be one of the causes, if not the only one, that leads to the loss of DA neurons and perturbation of DA system in zebrafish PD model of mutated *PARK2* gene.

#### PTEN-Induced Kinase 1 (*PINK1*) Gene

PTEN-induced kinase 1 (*PINK1*) gene encodes for PINK1 protein and is the second most common cause of autosomal recessive early onset PD after the *PARK2* gene (Ge et al., 2020). This protein is responsible for regulating mitochondrial quality control as it protects the neurons against mitochondrial dysfunctions by communicating with Parkin protein to perform mitophagy (Barazzuol et al., 2020). Other than that, PINK1 protein is highly sensitive to the presence of ROS and it plays an important role in the oxidative stress response mechanism (Barodia et al., 2017). PD patients with mutated *PINK1* gene were shown to have a significantly lesser amount of PINK1 protein, thus severing the PINK1/Parkin pathway, leading to mitophagy impairment and worse, neuronal apoptosis (Gandhi and Plun-Favreau, 2017).

Zebrafish *PINK1* gene is 57.8% identical to that of humans (Sallinen et al., 2010). Zebrafish with knockdown *PINK1* gene had defective mitochondria and exhibited developmental retardation (Anichtchik et al., 2008). Furthermore, reduction in the PINK1 population elicited oxidative stress to neuronal cells due to a substantial increase in ROS (Priyadarshini et al., 2013) and increased zebrafish susceptibility to MPTP neurotoxin (Sallinen et al., 2010). Besides that, PINK1-deficient zebrafish exhibited PD motor impairment symptomology of abnormal swimming behavior and poor response to tactile stimuli (Xi et al., 2010). In the context of DA neuronal loss, reported findings are rather controversial. Anichtchik et al. (2008) uncovered that the number of DA neurons was relatively decreased in PINK1-deficient zebrafish compared to the normal group. In contrast, a more recent study by Xi et al. (2010) revealed that knockdown of the *PINK1* gene in zebrafish only disturbed DA projections but did not result in substantial loss of DA neurons. These differences may be due to the different regions of interest investigated by each study; the former examined the posterior diencephalon while the latter reported on the ventral diencephalon. It is striking to investigate the causal effect of PINK1 deficiency that leads to the different observations seen between these two regions.

#### Presenilin-Associated Rhomboid-Like (*PARL*) Gene

The presenilin-associated rhomboid-like (*PARL*) gene is translated to a protease that localizes at the inner membrane of mitochondria (Lysyk et al., 2020). This protease is involved in mitochondrial function and is responsible for cleaving several proteins, including the PINK1 protein (Merhi et al., 2021). Notably, the PARL protease works upstream of the PINK1 protein. With this regard, PARL is considered a critical regulator of mitochondrial homeostasis (Liu et al., 2019). Nonetheless, it is important to note that the cleavage of PINK1 is also performed by several other proteases aside from the PARL protease (Noble et al., 2012). Although pathophysiological mechanism of the PARL protein is still elusive, mutations in the *PARL* gene have been linked to the development of familial PD (Noble et al., 2012). Also, the PARL protein regulates the degradation of damaged mitochondria via the PINK1/Parkin-dependent pathway (Kawamoto et al., 2020). Indeed, the PARL protein influences the PINK1/Parkin mitophagy pathway, and dysregulation to the pathway is one of the well-known pathogeneses of PD (Kawamoto et al., 2020; Merhi et al., 2021). Interestingly, Kawamoto et al. (2020) found a significant amount of PARL proteins accumulated inside the LBs of PD patients. The sequestration of these proteases into LBs further disrupted the PINK1/Parkin mitochondrial degradation pathway.

Zebrafish express two paralogs of the *PARL* gene, namely *PARLA* and *PARLB*. The former has 67% amino acid sequence similarity with humans while the latter is 55% similar (Shamchuk et al., 2017). Both the *PARL* paralogs are expressed in the embryos and adult zebrafish tissues (Noble et al., 2012). As seen in humans, zebrafish studies on the loss-of-function of the *PARL* genes results in DA neuronal perturbation and increased mortality, which further proves its influence in PD development (Shamchuk et al., 2017). Interestingly, knockdown of either one of the two paralogs did not result in reduced survival of the zebrafish, indicating a possible compensatory mechanism between the two genes (Noble et al., 2012). In a more recent study, the loss-of-function of the *PARLA* gene was found to decrease the expression of DA neurons primarily in the olfactory bulb (Merhi et al., 2021). The observation is supported by the poor performance of the mutant zebrafish in the olfactory test—reacted poorly to cadaverine (a repulsive stimulus). Besides, the same study also reported that the loss of *PARLA* function results in reduced distance traveled and velocity as well as an increased in freezing bouts during swimming, all of which indicate locomotor impairments (Merhi et al., 2021). All in all, although the mechanism of the *PARL* gene in inducing PD phenotypes is still largely unclear, animal studies have proven its influence in PD pathogenesis.

#### Parkinson’s Disease Protein 7 (*PARK7*) Gene

In humans, the expression of the *PARK7* gene is translated to DJ-1 protein. Mutations in the *PARK7* gene facilitate the development of early onset autosomal recessive PD (Hughes et al., 2020). DJ-1 protein plays an important role in normal human physiology as it is involved in transcription regulations of genes that are associated with oxidative response mechanisms (Ariga et al., 2013). DJ-1 helps the cell to survive oxidative stress by regulating the transcription of genes with antioxidant and anti-apoptotic properties (Wilson, 2011; Barnhill et al., 2020). Furthermore, inactivation of this protein was shown to induce the expression of P53 and BAX genes, both of which are mainly responsible for eliciting cell apoptosis (Ariga et al., 2013; Kato et al., 2013). Typically, symptoms such as young-onset motor disability, muscle rigidity, and tremors are seen in PD patients with inactivated DJ-1 protein (Repici and Giorgini, 2019).

The zebrafish *PARK7* gene is 83% identical to human DJ-1 (Selvaraj and Piramanayagam, 2019). Zebrafish expressing mutated DJ-1 protein exhibit characteristics that reflect PD motor symptoms in humans, such as reduced swimming velocity and increased freezing bouts. Furthermore, knockdown of the *PARK7* gene using morpholino oligonucleotides increased the production of ROS and consequently heightened DA neuronal susceptibility toward oxidative stress (Dolgacheva et al., 2019). According to Edson et al. (2019), the knockdown of the *PARK7* gene indirectly leads to the death of DA neurons not just because it leads to the increase of ROS population, but it also causes inhibition to proteasomal activity required for the mitophagy process. The functional annotations of DJ-1 protein have given clear insights into the importance of redox regulation in preventing cellular degeneration and maintaining cell survivability.

#### Leucine-Rich Repeat Kinase 2 (*LRRK2*) Gene

The leucine-rich repeat kinase 2 (*LRRK2*) gene mutations are the most common cause of late-onset autosomal dominant PD (Prabhudesai et al., 2016). This gene encodes for LRRK2 protein that is mainly involved in kinase activity. In normal conditions, LRRK2 protein mediates several physiological mechanisms such as protein autophagy and immune response. However, mutated LRRK2 proteins possess a higher degree of kinase activity than normal (Tsika and Moore, 2012; Rui et al., 2018). This phenomenon causes aberrant protein degradation, dopamine transmission disturbance, abnormal inflammatory responses, increased ROS production, and DA neuronal death (Tsika and Moore, 2012). Progressively, the uncontrolled kinase activity leads to the development of late-onset PD. Other than that, since the mutated protein is not able to perform autophagy like normal, it is believed to be one of the factors associated with α-synuclein protein accumulation (Martin et al., 2014) and LB inclusions (Thomas et al., 2020).

Zebrafish expresses LRRK2 protein that has 71% functional similarity to human LRRK2 (Sheng et al., 2010; Barnhill et al., 2020). The function of LRRK2 protein in both humans and zebrafish is said to be highly conserved as the mutations in zebrafish LRRK2 also cause neuronal cell loss and synuclein aggregation, similar to those seen in humans (Prabhudesai et al., 2016; Seegobin et al., 2020). Furthermore, a study conducted by Sheng et al. (2010), reported that deletion of a specific domain in LRRK2 (WD40 domain, known to regulate LRRK2 kinase activity) of the zebrafish model induces diencephalic DA neurodegeneration and reduces swimming distance (indicating movement impairment). Treatment with LRRK2 mRNA over-expression reciprocated the observations (Sheng et al., 2010). Finally, Sheng et al. (2018) in a more recent study described the effect of LRRK2 mutant zebrafish on heightened kinase activity and weakened immunity toward bacterial infection (Sheng et al., 2018). From the above findings, it can therefore be understood that the LRRK2 protein is important for protein degradation as well as redox and immune regulations. Mutations to the *LRRK2* gene cause gain-of-function to the protein and trigger the risk of developing late-onset PD.

Throughout the development of zebrafish as a model organism, these physiological and behavioral changes have provided important indicators that differentiated normal and parkinsonian zebrafish. It is striking to note that, like the rodent model, optimized and standardized behavioral assessments are needed to increase the accuracy and reliability of the zebrafish model (Vaz et al., 2018), in parallel with its advancing application in PD studies. Table 2 below summarizes the observed effects of neurotoxin inductions and PD-associated gene mutations on the zebrafish model of PD.

## Conclusion and Opinions

The era of translational medicine has become one of the greatest contributors to the scientific world. Danio rerio in particular, has provided a new platform, particularly in neurodevelopmental research, that has led to valuable findings and discoveries. Several neurodegenerative diseases such as PD, Alzheimer’s disease, and Huntington’s disease have benefited from this species. With its transparent embryos, easy genetic manipulations, and short life cycle, zebrafish have made molecular studies, particularly the omics, possible in a way that primates and rodents were unable to. Today, zebrafish are considered an excellent paradigm for the analysis of neurobehavioral dimensions of human relevance. It is also used in biology, neuroscience, pharmacology, and toxicology research. We have identified the efficacy and significance of zebrafish in this manuscript as a model for the screening of novel drugs for various neurological disorders.

Recent advancement in the pathological analysis of PD using zebrafish as a model has been reviewed in the current review. The gene knockdown of orthologous zebrafish or transgenic expression of pathogenic genes associated with human neurodegenerative disorders triggers key morphological, physiological, and biochemical defects in particular classes of neurons shared with other animal and human models. This indicates a substantial degree of functional conservation between human neurodegenerative disease-related genes and their orthological zebrafish. Thus, in elucidating the molecular basis of PD, zebrafish may be a good alternate model. Taking into consideration certain special characteristics of the zebrafish, we can foresee its expanded use as a high-throughput drug screening vertebrate platform. In parallel with the concept of precision medicine, ample knowledge on the omics (genomics, proteomics, metabolomics) of a disease can better help healthcare practitioners to tailor the treatment to individual patients. This patient-centered approach is believed to provide maximum treatment efficacy as it evaluates each patient individually. Since a majority of PD cases are reported to be sporadic (not inherited) (Klein and Westenberger, 2012), knowledge on precision medicine will be beneficial in determining the most suitable therapeutic strategy for each PD patient. While there is intensive and ongoing research investigating the etiology and pathophysiology of PD, researchers are still far from understanding this disease, especially at the molecular genomic and proteomic levels (Zolezzi et al., 2019). In achieving this, zebrafish have been, and will continue to be the ideal PD model, especially in studies where molecular investigations are needed.

## Author Contributions

KR wrote the article in consultation from all authors. WMYM conceived the idea and contributed to the overall structuring of the article, arguments, critical revision, and approval of the final version. NO, MHMN, AAD, JK, WNI, and NMI gave inputs, reviewed, and revised the article.

## Conflict of Interest

The authors declare that the research was conducted in the absence of any commercial or financial relationships that could be construed as a potential conflict of interest.
